# Methodology for Assessing Rice Varieties for Resistance to the Lesser Grain Borer, *Rhyzopertha dominica*

**DOI:** 10.1673/031.008.1601

**Published:** 2008-02-28

**Authors:** Y Chanbang, F. H Arthur, G. E Wilde, J. E Throne, B. H Subramanyam

**Affiliations:** ^1^ Chiang Mai University, Chiang Mai, Thailand; ^2^ USDA-ARS, 1515 College Avenue, Manhattan, KS, USA; ^3^ Department of Entomology, Kansas State University, Manhattan, KS, USA (retired); ^4^ Department of Grain Science and Industry, Kansas State University, Manhattan, KS, USA

**Keywords:** host plant resistance, rice varieties, Oryza sativa, rice type, *Rhyzopertha dominica*

## Abstract

Several physical and chemical attributes of rice were evaluated to determine which character would be best to use to assess multiple rice varieties for resistance to the lesser grain borer, *Rhyzopertha dominica* (F.). Laboratory tests were conducted on single varieties of long-, short-, and medium grain-rice to develop procedures and methodologies that could be used for large-scale screening studies. Progeny production of *R. dominica* was positively correlated with the percentage of broken hulls. Although kernel hardness, amylose content, neonate preference for brown rice, and adult emergence from neonates varied among the three rice varieties tested they did not appear to be valid indicators of eventual progeny production, and may not be useful predictors of resistance or susceptibility. Soundness and integrity seem to be the best characters to use for varietal screening studies with *R. dominica*.

## Introduction

Rice, *Oryza sativa* L., is a staple food grain for much of the world, and in some regions is a more important grain crop than either wheat or corn. Rice can be classified by the length-to-width ratio of the kernel; long grain, 3.4 (or more) to 1; medium grain, 2.3–3.3 to 1; and short grain, 2.2 (or less) to 1 (USDA 1994). There are many improved rice varieties of all three general grain types (Webb 1980; Childs 2004), and most of the main rice-growing regions throughout the world have had cooperative breeding programs for new varieties since early in the 20th century (Moldenhauer et al. 2004).

*Rhyzopertha dominica* (F.) (Coleoptera: Bostrichidae), the lesser grain borer, is a major insect pest of stored commodities, including rough rice, which is rice stored in bulk with the hull intact. The larval stage is spent inside the kernel, and both larvae and adults cause weight loss in kernels through feeding damage. Gundu Rao and Wilbur (1957) reported weight loss of about 9.5% in wheat kernels when larvae fed for 20 days, and weight loss of 19.4, 12.0, 9.5 and 6.5% per kernel during the first, second, third, and fourth weeks, respectively, after adult eclosion. Similarly, Nigam et al. (1977) compared weight loss from feeding of adult *R. dominica* in 10 varieties of rough rice, and reported damage ranging from 2.3 to 12.2%.

Some comparative studies have reported less damage caused by *R. dominica* on rice compared to wheat and other small grains (Baker et al. 1991). Also, the developmental period of *R. dominica* and oviposition rate can vary among different grain types and even within varieties of the same kind of grain. These differences have been used to generalize grain types as either tolerant or susceptible to *R. dominica* (Dobie and Kilminster 1978; Singh et al. 1986). Juliano (1981) states that hull integrity, kernel hardness, and chemical composition of the kernel may confer resistance to *R. dominica* and other insect pests, and references an earlier study by Breese (1960) which also discusses the soundness of the rice hull as a barrier to insects. However, neither of these studies quantitatively related the percentage of broken and cracked hulls in a rice variety with progeny production of *R. dominica*. The objectives of this study were to: 1) correlate the presence of broken and cracked hulls with progeny production of *R. dominica*; and 2) determine if either hull integrity or kernel characteristics would be useful characters to screen rice varieties for resistance to *R. dominica*.

## Materials and Methods

Three rice varieties were used in our assessments; Cocodrie, a long-grain variety obtained from the University of Arkansas, Fayetteville, AR, a medium-grain variety M-205 and a short-grain variety S-102, both obtained from Lundberg Family Farms, Richvale, CA. Upon arrival at the Grain Marketing and Production Research Center, Manhattan, KS, the rice was held at 4°C until used in the experiments.

For the initial tests to determine the percentage of cracked and split hulls, approximately 1,000 g of rough rice of each variety were cleaned by removing extraneous material through a #12 sieve (1.7 mm mesh openings), and the moisture content was determined using a Dickey-John moisture meter GAC 2000 (Dickey-John Corp., Auburn, IL, www.dickey-john.com). Tap water was added to each of the rice lots to bring all three varieties to a uniform moisture content of 14%. For each of 5 replicates, 20 grams of rice were sampled from the cleaned rice, and 1,000 hulls (with kernels inside) of each variety were microscopically inspected and classified as follows: solid hulls, with the palea and lemma intact and no space between the palea and lemma; split hulls, spaces down the longitudinal seam of the hull between the palea and lemma; and cracked hulls, the palea and lemma cracked longitudinally, but not on the seam or transversely across the hull. When hulls are split and cracked the brown rice is visible. The numbers of split and cracked hulls were combined and calculated as percentage of the 1,000 whole kernels that were examined.

Individual kernels of the three varieties were analyzed for kernel hardness by removing the hulls of 500 g of rough rice using a McGill Sheller, and then 300 of the brown rice kernels were randomly examined using the Single Kernel Characterization System (SKCS 4100, Perten Instruments, Springfield, IL, www.perten.com) to classify kernels according to hardness. If the means for kernel hardness were > 46 the sample was classified as hard, if the means were ≤ 46 the sample was classified as soft, following procedures described by Maghirang and Dowell (2003). Approximately 25 g of brown rice of each variety was weighed and ground to flour by using a Wiley Mini Mill (Arthur H. Thomas Company, www.thomassci.com) and sieved through a # 40 sieve (0.43 mm openings). Amylose content was determined using an amylose / amylopectin assay kit from Megazyme® (Megazyme International Ireland Ltd., www.megazyme.com). This is a standard method used to determine amylose content, modified from Gibson et al. (1997), which measures amylose content without prior starch purification and allows for variation in sample size. Data for percentage of split and cracked kernels, kernel hardness, and amylose content were analyzed using the General Linear Model (GLM) procedure of the Statistical Analysis System (SAS Institute 2001, www.sas.com) to determine significant differences among rice types at a level of *P <* 0.05.

Voucher specimens of *R. dominica* used in this study came from a laboratory colony, and specimens from this colony have been deposited in the Kansas State University Museum of Entomological and Prairie Arthropod Research as voucher specimen number 162. Progeny production was evaluated by placing 20 g of each variety of rice in separate 29-ml vials, and placing 20 unsexed 1–2 week-old adult *R. dominica* inside each vial for two weeks. All vials were placed in plastic humidity boxes (Arthur 2000) containing a saturated sodium chloride (NaCl) solution to maintain relative humidity at 75% (Greenspan 1977), which is equivalent to about 14.0–14.5% grain moisture content, and then incubated at 32°C. After 2 weeks, the rice in the vial was sieved, adults were removed, and the rice and all frass material were put back into each vial. The vials were returned to the incubator, and after 8 weeks the rice was sieved again, and the adults tabulated. Each trial was repeated five times at intervals of two weeks for ten weeks. Raw data for F1 progeny were transformed by square root, and data were analyzed by using the MIXED Procedure (SAS Institute, 2001) and means were separated using the Tukey-Kramer test at *P* < 0.05. The data for split and cracked kernels were assessed for correlation with progeny production using the Correlation Procedure in SAS.

As part of this study, the preference of neonate *R. dominica* for the three varieties of rice was also assessed. Neonates were collected by first placing 500 adult *R. dominica* into a 0.95 L glass jar with 500 g of rough rice and holding this jar at 27°C and 68% relative humidity. After two days, the adults were removed, and after an additional 8–9 days, neonates were collected from the jar by sieving the rice through a #30 sieve (0.6 mm openings). The neonates were individually removed from the frass by putting the collecting dish under a microscope, removing the neonates from the dish using a damp artists' paintbrush, and transferring each neonate into an individual well of a 24-well plate. Each well contained two brown rice kernels of each variety (6 kernels total in each well). The brown rice kernels were placed alternately along the well bottom as a ring in each well of a 24-well plastic plate (diameter of an individual well in the plate was 150 mm). The brown rice kernels were stuck to the bottom of the well using tapioca glue, which had been prepared by mixing tapioca flour in water (1 mg of flour per 2 ml of water) and heating until the solution dissolved into a paste. The glued kernels were allowed to dry for about 3 hours, and then one neonate was introduced in the middle of each of the individual wells comprising the well plates. Five separate replicates were performed as blocks at bi-weekly intervals. All of the well plates were stored inside an incubator at 32°C and 75% relative humidity as previously described. Five weeks after the neonate introductions, the well plates were examined under a stereomicroscope for the presence of an adult and an emergence hole in one of the kernels. The number of adults emerging from each rice variety was analyzed by using the General Linear Model procedure of SAS.

## Results and Discussion

More progeny were produced in Cocodrie variety rice compared to M-205 (Table 1) (*F* = 9.5; *df* = 2, 8; *P* < 0.01), while the percentages of split and cracked hulls in both Cocodrie and S-102 were greater than the percentage of split and cracked hulls in M-205 (*F* = 7.9; *df* = 2, 12; *P* < 0.01). In addition, there was a positive correlation between split and cracked hulls combined and progeny production (*r* = 0.57, *P* (r > 0) = 0.02). Kernels from all three rice varieties were classified as hard using the SKCS system, and each variety was significantly different from the others, however, there was little variation about the means for each of the varieties (Table 1). *R. dominica* showed no preference among the brown rice from the three varieties (*F* = 2.5; d.f. = 2, 12; *P* = 0.12), and the number of emerged adults from the 24 neonates exposed on the brown rice was 6.6 ± 0.7, 4.0 ± 0.7, and 6.8 ±1.4 in Cocodrie, M-205, and S-102, respectively. Amylose content was greatest in Cocodrie variety rice (Table 1), but amylose content was not correlated with progeny production (P ≥ 0.05).

**Table 1.**
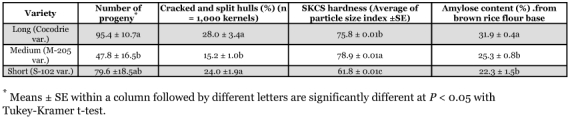
Number of F1 progeny, the percentage of cracked and split hulls, the hardness of the kernel, and the amylose content in three rice varieties.

The integrity of the rough rice hull appears to be a major factor in susceptibility of individual kernels to *R. dominica*. In these tests, there was a positive correlation between progeny production and the percentage of broken hulls. Breese (1960) and Cogburn (1974) also noted that the hull offers protection from stored-product insects, and, therefore, hull integrity is a valid measure of resistance or susceptibility. When the rough rice hull is removed, the brown rice is vulnerable to attack by neonate *R. dominica*. Progeny production in brown rice is consistently greater than progeny production in rough rice of the same variety (McGaughey 1970; Sittisuang and Imura 1987).
